# Towards Generalized Bioimpedance Models for Bladder Monitoring: The Role of Waist Circumference and Fat Thickness

**DOI:** 10.3390/s25247635

**Published:** 2025-12-16

**Authors:** H. Trask Crane, John A. Berkebile, Samer Mabrouk, Nicholas Riccardelli, Omer T. Inan

**Affiliations:** 1 School of Electrical and Computer Engineering, Georgia Institute of Technology, Atlanta, GA 30308, USA; jaberkebile@gatech.edu (J.A.B.); samer92@gatech.edu (S.M.); omer.inan@ece.gatech.edu (O.T.I.); 2 Urology and Critical Care Division at Becton, Dickinson and Company, 8195 Industrial Blvd, Covington, GA 30014, USA

**Keywords:** bladder, subcutaneous fat, electrical impedance, volume estimation, waist size

## Abstract

Continuous bladder volume monitoring in a wearable format can improve outcomes for patients with bladder dysfunction, heart failure, and other conditions requiring precise fluid management. Bioimpedance-based methods offer a promising, noninvasive solution; however, the influence of patient-specific anatomy, particularly waist circumference and subcutaneous fat thickness, remains poorly characterized. In this study, we use *in silico* finite element modeling to quantify how these anatomical factors affect two key bioimpedance metrics: voltage change (ΔV) and voltage change ratio (VCR). Comprehensive simulations were performed across 15 virtual anatomies, generating a reference dataset for guiding future analog front-end and algorithm designs. We further compared generalized volume estimation models against conventional patient-specific void regression approaches. With appropriate input scaling, the generalized models achieved performance within 10% of patient-specific calibrations and, in some cases, surpassed them. Certain configurations reduced mean average error (MAE) by more than 20% relative to individualized models, potentially enabling a streamlined setup without the need for laborious ground-truth acquisition such as voided volume collection. These results demonstrate that incorporating simple anatomical scaling can yield robust, generalizable bladder volume estimation models suitable for wearable systems across diverse patient populations.

## 1. Introduction

Accurate bladder volume monitoring is important in a variety of clinical settings, from critical care units to outpatient environments. Clinicians rely on bladder volume and urine output measurements to assess fluid balance, renal function, and the effectiveness of therapies such as diuretics [1,2]. Patients with neurological impairments, including those lacking normal filling sensations, often require timely notification for voiding to prevent overdistension and potential renal complications [3,4,5]. Even in routine outpatient care, urine output tracking can alert both patients and healthcare providers to early signs of fluid imbalance and guide interventions [6,7,8]. However, in both inpatient and home settings, current approaches to bladder monitoring raise concerns regarding infection risks, particularly with indwelling devices, or demand significant patient adherence, creating potential barriers to consistent, long-term usage [6,9].

Current standards for accurate urine output monitoring for inpatient settings are indwelling urinary catheters. Although they allow continuous bladder drainage and direct volume measurement, catheters introduce infection risk, resulting in one million urinary tract infections annually in the United States [9]. Notably, 52.8% of days with catheter use in elderly patients have been found to be inappropriate; the catheter was placed simply for the convenience of care while the patients were ambulatory or had a bedside commode available [10]. The convenience of the catheter-based approach to the clinical care team must be weighed against the costs of infection, estimated to be between 115 million and 1.82 billion dollars annually [11]. To reduce infections, costs, and unneeded catheterizations, noninvasive bladder monitoring methods have become a priority in healthcare innovation.

One noninvasive modality used clinically today is ultrasound, which requires a trained clinician to perform periodic scans using general-purpose tabletop ultrasound devices. Such devices require a medical worker to perform each scan and have a MAE of 21.8%, which is highly influenced by the technician’s expertise and the accuracy of the scanner placement [12]. Handheld ultrasound bladder scanners, such as BVI 9400^™^ and Prime^™^ from BladderScan^®^, offer smaller errors [11] but still do not support fully continuous monitoring. Wearable ultrasound systems have been proposed to increase scan frequency, yet focus only on detecting voiding [13,14] or bladder state (full vs. empty) for incontinence management, rather than continuous volume estimation [15].

Another potential modality in earlier stages of development is near-infrared spectroscopy (NIRS) technology, which uses the absorption of varying visible and infrared light to quantify the bladder’s fill state [11]. Work has mainly focused on using NIRS to assess bladder dysfunctions rather than predicting bladder volume [16,17]. Of the few works that have focused on bladder volume, the ability to tell low, ≤250 ${cm}^{3}$, and high, ≥300 ${cm}^{3}$ volumes is possible [18]. Prediction of actual bladder volume using state-of-the-art machine learning models yields MAE of 113.4 mL or 22.6% [19], comparable to non-specialized ultrasound devices. With few studies in this area, more work is needed to improve consensus on methods and accuracy for NIRS in clinical bladder volume monitoring applications.

A modality not yet clinically used, but holding merit for accurate volume estimation in a wearable form factor is electrical bioimpedance, specifically comprising bioimpedance analysis (BIA) and electrical impedance tomography (EIT) approaches. The technology works by injecting a small, safe, imperceptible current into the body and measuring the resultant voltage via electrodes [20]. BIA takes this measurement from one frame of reference. The resultant voltage change expressed as a ratio to a baseline measurement, known as the *VCR*, has been demonstrated to correlate well with bladder volume [21,22,23]. These systems can be made into wearables for continuous monitoring, unlike ultrasound, which requires a trained individual to perform a spot check [11,23]. Additionally, BIA can achieve better in vivo MAE of 63.0 mL compared to NIRS 113.4 mL error. While bioimpedance has strengths compared to other methods, it can be sensitive to changes in the tension of the abdominal muscles, leading to potentially increased errors [23].

Relatively little attention has been paid to patient-specific factors, particularly waist circumference and subcutaneous fat thickness, and how they affect signal integrity and volume estimation accuracy. It has been hypothesized and demonstrated that adipose tissue can attenuate the bioimpedance signal, altering the measured impedance. Specifically, in vivo and in silico measurements have found adipose tissue attenuates the observed bioimpedance signal, altering the measured impedance [22,24,25]. Additionally, variations in the patients’ waist size change the distance between electrodes and the bladder, further contributing to intersubject variation of observed impedance values due to volume change [22]. These anatomical variations can reduce the effective dynamic range captured by the analog front end (AFE), impacting the sensitivity of bladder volume estimation across the full filling cycle and the fidelity of downstream EIT conductivity reconstructions.

EIT is performed by taking BIA measurements from varying electrode positions to reconstruct a tissue conductivity map [20,26,27]. In contrast to single-frame BIA, which can correlate strongly with bladder filling but cannot localize the source of the fluid signal [21,22,27,28], EIT leverages multiple measurement frames and *a priori* knowledge of the skin boundary to estimate spatial conductivity distributions. These measurement frames are typically sequenced using the neighboring method of electrode addressing, with a single row of electrodes [29]. Such an addressing scheme is not optimized to maximize bladder sensitivity, but rather to achieve an equal spatial distribution of sensitivity throughout the cross-section [30,31]. Simulation studies using EIT with varying electrode configurations have shown that multi-row arrangements, such as 2 × 8 or 4 × 4 layouts, yield the highest global impedance, defined as the sum of all pixel values in the reconstructed conductivity image [29]. However, relying on global impedance as an estimator is prone to error. Changes in conductivity outside the bladder region, like patients breathing or movement, also alter the summed value, introducing confounding components into the signal [32]. Prior work has demonstrated that even at an SNR of 40 dB, global impedance-based estimators can exhibit relative errors exceeding 200% [33]. Works in vivo measuring bladder volume 30 s before void showed relative errors of (32±18)% at high bladder volume. When predicting residual volume, measured 30 s after void, the errors were (72±58)%, demonstrating that this method’s accuracy deteriorates at low volumes [25]. Other EIT approaches have shown that in vivo bladder volume estimation errors at void can be reduced to less than 8% when reconstruction is performed in 3D rather than 2D, with volume derived from the integrated voxel volume of high-conductivity regions limited to where the bladder could be located [34]. Progress in other EIT applications shows that techniques such as 3D reconstruction, deep learning frameworks, and redesigned electrode layouts can meaningfully advance performance, suggesting promising avenues for improving bladder-volume estimation as well [35,36,37,38,39]. These works have largely prioritized algorithm development and the miniaturization of data-acquisition hardware for wearable applications but have yet to assess the impact of patient-specific anatomical variability.

**Figure 1:**
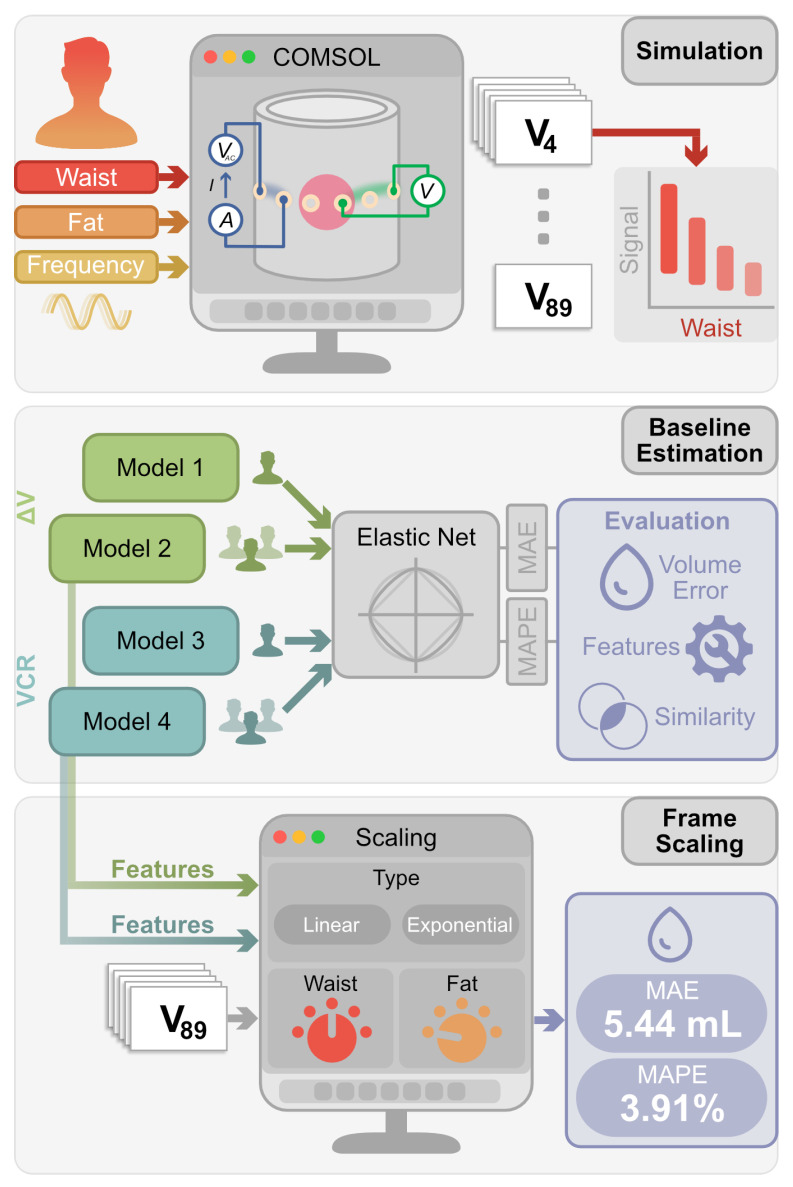
Overview of study design. The simulation environment iterated over all combinations of waist circumference, subcutaneous fat thickness, and stimulation frequency to compute voltages for 90 unique measurement frames. The data was analyzed to assess the impact of these input characteristics on signal behavior and model performance. Baseline estimations included patient-specific models and a naïve generalized model that directly incorporated patient characteristics as features. Two scoring criteria, MAE and mean average percent error (MAPE), were used to evaluate error, feature sparsity, and feature selection similarity. To improve the generalized models, linear and exponential scaling methods were applied to the measurement frame inputs before volume estimation.

There is a pressing need for an understanding of generalized bioimpedance models that account for a range of patient anatomies, including variation in waist size and subcutaneous fat. In this paper, we (1) quantify how these anatomical characteristics influence voltages in BIA-based bladder monitoring and (2) compare linear and nonlinear normalization methods to enable volume estimation algorithms to generalize across patient anthropometrics, as overviewed in Figure 1. Using COMSOL Multiphysics^®^ v6.0 simulations [40], we demonstrate how subcutaneous fat thickness and waist circumference drive significant differences in the measured signals, influencing the performance of volume estimation. To help facilitate broader adoption and wearable design optimization, we provide reference tables detailing these variations, empowering researchers to scale their existing data to validate their methods against diverse patient populations. Ultimately, by incorporating patient-specific considerations into bioimpedance modeling, we seek to improve bladder volume measurement accuracy to address critical needs in managing fluid balance for congestive heart failure and other patient groups. This work supports future efforts to develop robust, calibration-free monitoring systems that can operate reliably across heterogeneous user populations, thereby increasing clinical utility and reducing the burden of individualized setup.

## 2. Materials and Methods

### 2.1. Simulation Design

To evaluate how subcutaneous fat and waist circumference affect bioimpedance signals, we designed a conductivity simulation model in COMSOL with the AC/DC physics module (COMSOL AB, Stockholm, Sweden). We extended our previously validated digital twin framework [41] by modifying the domain geometry to enable automated parameter sweeps of patient-specific characteristics. The model represents the abdomen as a circular cylinder with a fixed height of 30 cm and variable circumference. This approach maintains low computational cost and is a commonly accepted practice of using a cylindrical model as simulation results have been shown to transfer sufficiently well to in vivo conditions [22,33,42].

The skin domain formed the outer surface of the model, with a thickness of 0.15 cm derived from reported dermal measurements [43]. Boolean operations were used to isolate a 2 cm-radius region of skin centered at each electrode location and positioned vertically at the model midline. This reduction in the modeled skin’s surface area substantially decreased the degree of freedom (DOF) required for simulation while preserving voltage accuracy, as verified through convergence testing. Six electrodes, each with a radius of 1.5 cm, were positioned on the skin surface in an equiangular arrangement, with the final electrode placed diametrically opposite the first on the anterior half of the model. The electrodes were formed from the skin’s cylindrical shell using Boolean operations. The process resulted in a uniform electrode thickness of 0.5 cm. This six-electrode configuration was chosen as a practical balance between spatial resolution and computational efficiency, providing sufficient coverage for multiple current–voltage pair combinations while keeping the total number of simultaneous physics nodes solvable within the available memory resources.

Beneath the skin domain lies the subcutaneous fat layer, represented as a cylindrical shell. The model’s interior comprises two regions: the background tissue and a variable-volume bladder represented as a sphere. The background tissue was assigned the dielectric properties of muscle, since most abdominal organs consist primarily of muscular tissue or exhibit similar dielectric characteristics in both males and females [22,44,45]. The bladder was positioned at the model center with a fixed anterior offset of $\frac{1}{3}r_{\mathrm{waist}}$ and a variable diameter corresponding to the simulated bladder volume. We selected this offset based on our previous observations segmenting MRI images, such that as the bladder expanded, it would grow towards the abdominal wall without intersecting with the subcutaneous fat layer [41,46].

To enable a fully automated simulation workflow, all unique electrode pair combinations for current injection were predefined, resulting in 62=15 independent electric current physics nodes within the COMSOL component. The approach allowed for the comprehensive exploration of our swept parameters of interest without requiring external scripts to modify the current electrode selection. The swept parameters under consideration are detailed in Table 1. The waist circumference used six values that are inclusive of the 10th to 90th percentile of U.S. adults aged 20 years and older [47]. The subcutaneous fat layer was parameterized using four thickness values encompassing the range observed in adults across all BMI categories [48]. Selected bladder volumes included those that cover the 95% confidence interval (CI) range for bladder fullness grades 0 to 3 at void: 10, 20, 100, 220, 340 and 460 mL [49]. A multi-frequency study used five steps per decade, incorporating standard frequencies typically employed in BIA and EIT research, alongside several higher frequencies for a total of nine values [15,27,50]. For all simulations, a current of 1 mA was driven between the current carrying (CC) electrodes.

The frequency-dependent dielectric properties of the domains are specified in Table 2. The study referenced for gel electrode values did not report Cole-Cole parameters but did report relative permittivity ($\varepsilon_{r}$) and conductivity (σ) in a graph [51]. We manually transposed the data into COMSOL and used interpolation to closely approximate the reported data. For all other domains, we found studies that reported 4-pole Cole-Cole parameters [51,52,53,54]. Additional details of the process of encoding these properties in simulation can be found in our previous work [41].

### 2.2. Signal Processing

Data was extracted using domain probes to obtain electrode voltages for each simulation configuration. For a given configuration, the COMSOL output forms a matrix $V^{6\times 15}$, where rows correspond to frequencies and columns to CC electrode pairs. For each CC configuration, voltage was measured across all possible non-CC electrode pairings, yielding 42=6 measurement frames per current injection. With 15 distinct CC configurations, this results in a total of 15×6=90 unique measurement frames, excluding reciprocals. All calculations were performed in Python 3.12 using the pandas package, a widely adopted tool for data analysis and manipulation [55]. The process was repeated across all model parameter configurations, generating a single pandas dataframe containing all experimental results. During the simulation process, some configurations may result in the bladder domain intersecting with the subcutaneous fat layer. Such cases cannot occur in vivo, as the bladder may only press against the fat layer, not breach such tissue [46]. Therefore, these impossible configurations were removed from the final analysis. The remaining configurations can be found in the Supplementary Material.

Two signals of interest were computed from the dataframe: ΔV and *VCR*. For both signals, the baseline voltages were determined by the 10 mL bladder volume simulations. While there is no consensus cutoff for clinically meaningful residual volumes, many works agree that ≤30 mL can be considered in normal ranges [56]. The calculation of *VCR* was defined by Shida et al. [21] as

(1) $$VCR=\frac{\left|V-V_{0}\right|}{V_{0}}\;\cdot \;100$$

where $V_{0}$ is the voltage at the baseline bladder volume and *V* is the voltage at some arbitrary volume for a given measurement frame. This method of calculation gives the change as a percentage and provides a level of normalization of the bioimpedance signal as the bladder accumulates urine. Similarly, $\Delta V=V-V_{0}$ but has no built-in normalization method. Both signals are used in BIA methods [21,22,23,42] and utilization of a baseline via ΔV is frequently used in EIT applications, especially in global impedance based, equivalent circular diameter, and other methods requiring calibration [11,25,33,34,57,58].

### 2.3. Baseline Volume Estimation Algorithms

The BIA and EIT literature has focused on patient-specific models using *VCR* or ΔV where a calibration is performed through linear regression against measured urine output. Ground-truth volume is typically found via commode or ultrasound on the first void [11,25,34]. To provide a performance benchmark, we implemented models following this conventional patient-specific calibration approach. The features used for the estimations were the 90 measurement frames. We selected elastic net as our regression model for its ability to balance promoting sparsity and regularization [59]. We applied leave-one-out (LOO) cross-validation on the measurements to appropriately assess performance. We investigated |ΔV| features first in virtual patient-specific estimators and then generalized to one estimator. These approaches are labeled as model one and two respectively. We repeated the process for |VCR| based features for models three and four. A summary of the modeling approaches is presented in Table 3.

The generalized baselines, models two and four, used a simplistic approach to generalization by providing waist size and fat thickness as additional features during training. Cross-validation used a *k*-fold approach configured as leave-one-out at the virtual-patient level, rather than at the level of individual measurements in the previous models. For all models, the solver’s maximum iterations were set to 100,000.

All models selected two sets of hyperparameters based on the scoring metric: MAE, and MAPE. Evaluation of the models considered the respective errors, number of features, proportion of features used in the neighbor set, and consistency of selected features across cross-validation folds. The neighbor set N are the measurement frames used in the neighboring method of selecting electrodes, the typical method in EIT literature [11,27]. The neighboring method dictates that only adjacent electrode pairs can be used. CC pair injects current, and voltages are sequentially recorded by all neighboring pick-up (PU) pairs. Once all combinations are completed, the CC increments to the next position and the process repeats.

The Jaccard index is a widely used similarity measure that quantifies overlap between two sets [60]. We adapted the index formula to assess the global agreement across cross-validation folds. Specifically, the mean pairwise Jaccard index $\bar{J}$ is defined in (2), where $S_{i}$ and $S_{j}$ denote the sets of features selected in folds *i* and *j* respectively, and *N* is the number of folds. The metric provides an interpretable measure of feature stability; it ranges from zero, no shared features between any fold pairings, to one, identical feature sets between all fold pairings.

(2) J¯=1N2∑i<j|Si∩Sj||Si∪Sj|

The second feature selection consistency metric used is stability. Stability quantifies how consistently individual features are selected across multiple random subsamples [61]. For each feature *k*, we compute its selection probability ${\hat{\Pi }}_{k}$, defined in (3), as the proportion of folds in which the feature’s coefficient is nonzero. The coefficients ${\hat{\beta }}^{\left(n\right)}$ were obtained from the trained elastic net model.

(3) $${\hat{\Pi }}_{k}=\frac{1}{N}\sum_{n=1}^{N}I\left({\hat{\beta }}_{k}^{\left(n\right)}\neq 0\right)$$

We then defined the set of stable features ${\hat{S}}^{\mathrm{stable}}$ as those whose selection probability satisfies $\pi_{thr}\geq 0.8$, a commonly accepted threshold [61], as shown in (4). The metric allows us to measure robustness to variation in the training data.

(4) $${\hat{S}}^{\mathrm{stable}}=\left\{k:{\hat{\Pi }}_{k}\geq \pi_{\mathrm{thr}}\right\}$$

### 2.4. Frame Scaling

Including waist circumference and subcutaneous fat thickness as direct model features did not improve generalized performance, as these parameters primarily influence the physical scaling of the measured voltages rather than contributing independent predictive information. To better account for anatomical variability, we implemented a frame-based scaling approach in which a single normalization factor is applied uniformly across all voltage features within each measurement frame. This factor adjusts the overall magnitude of the signal according to subject geometry while preserving the relative spatial relationships among electrodes.

We evaluated two scaling approaches: linear and exponential. The former has the lowest computational complexity, as lowering the complexity of estimation is a primary motivation, and serves as a scaled baseline. The exponential method, by contrast, is physics-informed, reflecting the exponential decay of electric field strength with increasing electrode separation due to larger waist circumference in the simulation domain [20]. The two scaled measurement sets were evaluated independently in our analysis. All models were trained and evaluated using LOO cross-validation, in which one patient’s configuration was excluded from training during each fold. The linear scaling formulation is defined as:

(5) $$V_{s}=\frac{V}{\alpha w\;\cdot \;\beta f}$$

Here $V_{s}$ is the scaled signal, *V* is the signal, *w* and *f* are the simulation geometry waist size and subcutaneous fat thickness, respectively, units are meters. The coefficients α and β were initialized using regression values derived from the simulation parameter study and subsequently refined through nonlinear curve fitting, using the subset of features selected by elastic net. The exponential scaling method is defined as:

(6) $$V_{s}=V\;\cdot \;\eta e^{-\delta_{1}w-\delta_{2}f}+\alpha$$

In this formulation, $\eta ,\;\delta_{1},\;\delta_{2},\;\alpha$ are scaling parameters learned through nonlinear regression, again using the elastic net-selected feature set. This approach captures the exponential attenuation behavior observed in bioimpedance physics [20], allowing the scaled signal to more accurately reflect geometric differences across patient anatomies. All curve fitting was performed using the optimize module in the SciPy Python package [62].

## 3. Results

Our results are reported with standard notations where $\bar{x}=$ mean, σ= standard deviation, and ranges with a ± denote $\bar{x}\pm \sigma$.

### 3.1. Impact of Patient Characteristics

**Figure 2:**
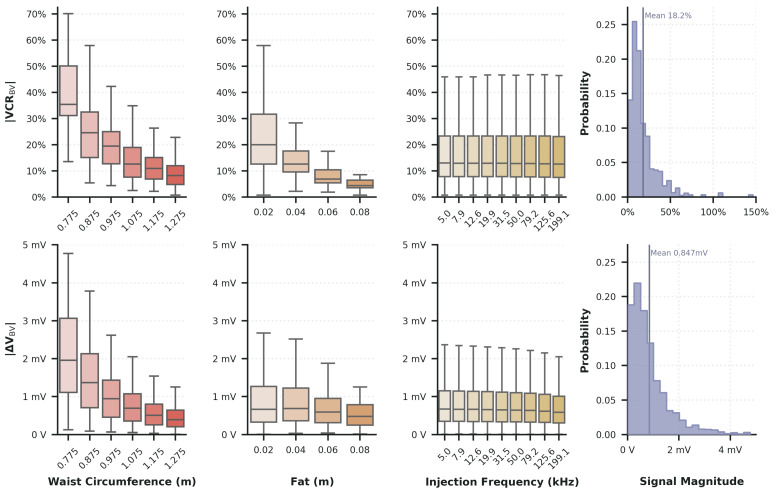
Overview of bioimpedance signal distributions with bladder volume fixed at the maximal simulated value. The observed spread reflects variability introduced by differing virtual patient characteristics. Simulation used 50 kHz unless otherwise noted.

Waist circumference was found to be the dominant anatomical factor influencing measurement sensitivity and signal amplitude, while fat thickness had a lesser, but still substantial effect at 50 kHz. The stimulation frequency in the range tested had comparatively minor effects. These parameters directly modulate current path length and tissue resistivity, thereby shaping the measurable impedance changes associated with bladder filling. Additional distributions of the observed values are shown in Figure 2. In these plots, bladder volume is fixed at the maximum simulated value, and the spread in the box plots reflects variability introduced by changes in waist circumference and fat thickness.

The bladder volume *VCR* ranged from 0.7% to 146.4% with a mean of 18.2% across all patient parameters at maximal bladder volume. Aggregated Pearson correlation coefficient (PCC) between bioimpedance and the waist, fat, and frequency parameters were −0.51, −0.37, and −0.01, respectively. The minimum waist circumference of 0.775 m observed a $|VCR_{BV}|$ range of (43.4±26.1)%, while the largest circumference of 1.275 m experinced a (10.0±7.6)% range across measurement frames. For the fat parameter, a similar attenuation of the signal occurred, with the minimum of 0.02 m yielding a range of $|VCR_{BV}|=$ (25.0±19.1)%, while the parameter maximum 0.08 m showed (6.6±6.3)%. This trend highlights how larger torso dimensions and increased adipose thickness reduce the current penetration in the region around the bladder, thereby compressing the dynamic range of the bioimpedance signal. Due to the low PCC of the stimulation frequency, the ranges of its minima and maxima are not reported.

The $|\Delta V_{BV}|$ signal ranged from 0.01 mV to 4.77 mV with a mean of 0.85 mV across all patient parameters at maximal bladder volume. The waist, fat, and frequency parameters had an aggregated PCC with bioimpedance of −0.59, −0.17, and −0.05, confirming the dominant influence of waist circumference between the two signals. For the minimum waist circumference, we observed a $|\Delta V_{BV}|=$ (2.06±1.22) mV, while the largest circumference exhibited a (0.44±0.28) mV across all measurement frames. The fat parameter’s minimum and maximum yielded (0.95±0.89) mV and (0.52±0.32) mV, respectively. For the stimulation frequency parameter, we found the lowest frequency had shown a range of (0.89±0.78) mV. The maximum frequency had (0.77±0.68) mV. The pronounced compression of both *VCR* and $\Delta V_{BV}$ with increasing waist circumference and fat thickness underscores their critical role in defining inter-subject variability and potential challenges of model generalization.

### 3.2. Baseline Volume Estimation

The baseline bladder volume estimation models’ performance is summarized in Table 4. Each model was evaluated under two separate error objectives: MAE and MAPE, resulting in two distinct fits per model. The patient-specific models, one and three, demonstrated the best performance for their respective data types, with lower errors and fewer selected features than the generalized models. Specifically, model three achieved the lowest error when fitting for both error objectives. When optimizing for MAE, the error was $\bar{x}=$ 1.65 mL at the cost of not performing any feature selection. In contrast, when using MAPE as the error objective, only 18.7% of features were used to achieve an error of $\bar{x}=$ 0.78%. The generalized models, two and four, showed increased errors but also higher feature selection stability relative to their patient-specific counterparts. Notably, model two showed a substantial improvement in stability, with the Jaccard index increasing from $\bar{J}=0.44$ to 0.89 and the stability score rising from 0.07 to 0.88, while selecting only 15.1% of available features. The *VCR* based models consistently used more features, including those in the neighboring method measurement frame set N, for both patient-specific and generalized models, compared to models that used $|\Delta V_{BV}|$.

Across all models trained with the MAE objective, the optimal α hyperparameter remained relatively stable around $1.00\times 10^{-7}$, as shown in Table 5. In contrast, α varied by up to three orders of magnitude across models trained with the MAPE objective, suggesting greater sensitivity to this error formulation. The $L_{1-\mathrm{ratio}}$ values for $|\Delta V_{BV}|$-based models exhibited low variability (≤0.1), whereas models based on *VCR* showed much larger fluctuations, with values spanning up to 0.6. Notably, *VCR*-based models also required significantly more time to train; approximately 60 times longer than their $|\Delta V_{BV}|$ counterparts due to sensitivity to scaling. Full cross-validation grid search error curves are available in the Supplementary Material.

### 3.3. Frame Scaling

The performance of generalized models under different input scaling methods is summarized in Table 6. Both model two ($|\Delta V_{BV}|$) and model four (*VCR*) were evaluated using feature sets selected under the two error objectives, MAE and MAPE, and tested with linear (5) and exponential (6) scaling strategies. Exponential scaling substantially improved performance across all conditions when compared to linear scaling. For Model 2, the MAE dropped from 35.49 mL to 8.78 mL, and the MAPE decreased from 24.17% to 9.31%, regardless of the feature selection objective. A similar improvement was observed for Model four, where exponential scaling reduced the MAE from 12.53 mL to 5.67 mL and the MAPE from 10.23% to 3.91% under the MAE-selected feature set. Out of the eight scaled input configurations tested, two achieved performance that surpassed at least one of the baseline patient-specific models.

## 4. Discussion

Conventional approaches to account for patient variability in bioimpedance-based bladder volume monitoring often compute an intermediate signal (e.g., global impedance, equivalent circular diameter), followed by a final linear regression step that serves as a patient-specific calibration [33]. This calibration requires a secondary measurement of bladder volume, either through indirect methods such as ultrasound or direct methods, such as collecting voided volume in a commode [11,25,34]. However, these calibration procedures introduce significant practical burdens: they require additional hardware, patient compliance, and can disrupt clinical workflows by necessitating tasks such as coordinating with medical staff when the patient can first void. These limitations hinder the scalability and ease of use of bioimpedance systems in real-world settings. This work systematically examines the influence of patient characteristics on bioimpedance measurements and introduces frame-scaling equations that mitigate these effects. The proposed approach supports the development of wearable bladder volume estimation systems that maintain accuracy across diverse anatomies and anthropometrics, thereby eliminating the need for cumbersome individual calibrations.

### 4.1. Patient Characteristics Impacts

Our results demonstrate that patient-specific anatomical features, particularly waist circumference and subcutaneous fat thickness, substantially influence both $|VCR_{BV}|$ and $|\Delta V_{BV}|$ signals. Both metrics exhibited a strong negative correlation with waist circumference and only weak dependence on stimulation frequency across the tested range. Subcutaneous fat thickness showed a moderate negative correlation with $|VCR_{BV}|$, but only a weak correlation with $|\Delta V_{BV}|$. These trends suggest that $|\Delta V_{BV}|$ may be a more robust signal for generalized model development, as it is less sensitive overall to variations in patient anatomy and may require less scaling to achieve accurate volume estimates.

The reported signal distributions across varying patient anatomies provide valuable insight for AFE and hardware design. As shown in Figure 2, the $|\Delta V_{BV}|$ and $|VCR_{BV}|$ plots quantify the expected maximum signal magnitudes and their variability due to anatomical factors. These results help establish practical bounds on AFE sensitivity and noise performance necessary to reliably detect volume changes across a diverse patient population. For example, designing an AFE capable of resolving bladder volume changes as small as 10 mL across all users requires accounting for the worst-case condition; that is, the smallest observed signal. One such case would be using the largest waist circumference; the mean voltage change was $|\Delta V_{\bar{x}}|=0.44\;mV$ for a bladder volume of 460 mL. To achieve the 10 mL resolution target, the system must therefore detect voltage steps of 9.56 μV or smaller. A complete AFE design is not included in this work, as the choice of topology, filtering, and amplification stages is highly dependent on the engineer’s objectives and the desired characteristics of the signal-acquisition circuitry. However, our results provide constraints on the allowable gain, input range, effective number of bits, full scale voltage range, and noise floor of the analog front end. If we wanted a more conservative estimation, we could use the first quartile value instead of $\bar{x}$. By providing a population-wide signal envelope, our results allow hardware developers to validate and optimize system performance for real-world variability without requiring extensive in vivo data collection.

Another important application of this characterization is in interpreting and normalizing simulation-based signal expectations across different body types. For example, in a study by Li et al., a single 2D torso model was used to identify optimal electrode placement based on the $|VCR_{BV}|$ feature, followed by validation in a human subject study with n=8 participants. Their simulation predicted a preferred location with $|VCR_{BV}|\;=63.9\%$; however, the same location yielded a mean in vivo value of only (27.5±14.7)% [22]. This discrepancy is likely due to anatomical differences, specifically increased adipose tissue in the experimental model compared to the simulated model, as discussed by Li. Specifically, the in silico model appears to be derived from a thin individual with minimal subcutaneous fat, whereas their participant cohort included a broader range of body types. In our simulations, increasing fat thickness and waist circumference from the nominal thin body habitus to the upper bounds of our study reduced the $|VCR_{BV}|$ signal by up to 73.4%, which encompasses the difference between Li’s single-subject simulation (63.9%) and their measured cohort mean (27.5%). The spread of $|VCR_{BV}|$ values generated by varying these parameters spans the range observed clinically, indicating that anatomical variability alone can explain the attenuation Li reported. Therefore, our results provide a quantitative signal envelope that can serve as a normalization reference to reconcile simulation and in vivo results.

### 4.2. Baseline Volume Estimation

Prior work relies on patient-specific calibrations, which serve as a black-box solution to the challenge of anatomical variability [11,25,34]. While these methods require individualized volume references, they can offer superior performance compared to generalized models when no feature scaling is applied. This trend was reflected in our results; patient-specific models achieved the lowest errors across both MAE and MAPE objectives, with model three reaching a 1.65 mL MAE and 0.78% MAPE. However, this high performance came at the cost of increased feature selection variability and the need for secondary calibration procedures. In practical terms, the measurement frames that optimized performance for one anatomical configuration were rarely selected in models trained on other anatomies. This finding underscores that patient-specific estimators do not generalize well: features relevant for one subset of patients often fail to translate to others. Consequently, studies relying solely on patient-specific calibration must include a broad range of anatomical types to avoid overfitting to a limited population. In contrast, the generalized models trade off some accuracy for much greater stability in feature selection. Most notably, model four with the MAPE criteria achieved errors of only 4.44% while achieving a Jaccard index and stability of 0.98. In the context of BIA and EIT systems designed for scalable, practical deployment, this tradeoff favors generalized models, which offer more interpretable and reproducible behavior across patient cohorts and within cross-validation folds in our study.

The behavior of the two data types, $|\Delta V_{BV}|$ and *VCR*, further emphasizes the practical tradeoffs in model design for bioimpedance-based bladder volume monitoring. While *VCR* based models incorporated a broader set of features, including the $F_{N}$, they showed greater sensitivity to hyperparameter tuning and took ≈60 times longer to fit. This sensitivity may stem from the inherent scaling introduced by the ratio, which amplifies measurements with very small initial conditions and can thereby distort the feature importance rankings. Future work could investigate normalization methods, such as taking the log of the *VCR*, to help with scaling issues.

Models that used $|\Delta V_{BV}|$ selected only a small proportion (≤0.16) of features from the neighboring measurement frame set, $F_{N}$. In contrast, prior EIT studies typically compute ΔV using only measurement frames within $F_{N}$ [25,34,42,63]. Our findings presented here build on previous work [41], which indicated that measurement patterns limited to the $F_{N}$ set may result in suboptimal signal characteristics, including reduced amplitude and diminished sensitivity to bladder volume changes.

### 4.3. Frame Scaling

We demonstrated that generalized models can be significantly improved beyond the naïve baseline, where patient characteristics are simply appended as features, by applying appropriate scaling on the measurement frames. Among the approaches evaluated, exponential scaling produced the most accurate estimations. This improvement is due to the underlying physics of BIA, where current density decays exponentially with distance from the source [20]. By weighting the frames proportionally to this decay behavior, the volume estimation model can compensate for various spatial sensitivity distributions due to physiological differences in anatomies. Importantly, the proposed framework relies on two simple anatomical metrics, waist circumference and subcutaneous fat thickness, that can be obtained quickly and noninvasively using a standard measuring tape and skinfold caliper [64]. The level of accuracy allowed generalized models to match, and in some cases surpass, the performance of patient-specific models reported earlier, while preserving the advantages of generalizability and reduced calibration burden. For instance, model four with exponential scaling achieved the highest overall performance, with an MAE of 5.44 mL and a MAPE of 3.91%.

From an implementation perspective, the proposed frame scaling procedure is well-suited for integration into real-time, low-power embedded systems used in wearable bladder monitoring. Specifically, from our previous work we have shown that direct volume estimation can be performed within 5 μs on an ARM Cortex-M0 microcontroller [41]. The additional work of frame scaling requires more multiplications to be performed, which will increase computation time and energy consumption slightly. From our previous wearable work with bioimpedance, one could expect such a system to have a battery life of over four days with a 500 mAh lithium polymer battery [65]. A single normalization factor, calculated from Equation (5) or Equation (6), is applied across all measurement frames, resulting in minimal computational burden and requiring only basic arithmetic operations. The anatomical parameters used for scaling can be measured once from the patient, and the scaling factor can be calculated during device setup and stored as constants. This eliminates the need for repeated calibration or user intervention, as the values would only need to be updated if the individual’s anatomy changed significantly. Moreover, the scaling operation maintains the spatial relationships among voltage frames, enabling it to be implemented immediately before the regression or neural inference stages without affecting the model architecture. As such, frame scaling can function as a lightweight preprocessing layer that improves model robustness to inter-subject variability while maintaining compatibility with existing impedance acquisition hardware and embedded firmware constraints. This design consideration supports the feasibility of continuous bladder monitoring in wearable form factors.

While this study offers new insights into the effects of patient characteristics on bioimpedance signals for bladder volume estimation, a computer model has limitations that open several directions for future work. Unlike our previous digital twin [41], which closely reflected in vivo tissue geometries, this study used a simplified cylindrical model that enabled complete control over patient-specific parameters and reduced computational complexity. The cylindrical modeling method introduces slight differences in the current density and therefore sensitivity to the bladder compared to realistic geometry. Prior work has used simpler 2D models, even without capturing the anatomical complexity of the torso—such as lateral curvature of the torso, hip structure, or tissue heterogeneity—to find viable electrode configurations that are successful when validated in vivo [22,25,34]. While this work is limited by the lack of in vivo validation, our simulations remain more comprehensive than those in previously successfully validated studies.

We designed the simulation to minimize build time by instantiating all CC electrode pairs as independent physics nodes in COMSOL to be solved simultaneously. Simulation approaches are inherently limited by available memory, which allowed for the simulation of six electrodes, corresponding to 15 independent CC pairs, at high mesh fidelity within the 32 GB memory of the desktop. This configuration provided a sufficient number of unique measurement frames to capture spatial sensitivity patterns while keeping computational cost manageable. Studies can feasibly test only a finite number of permutations; in this work, waist size, fat thickness, and bladder volume were evaluated across at least 80% of the observed empirical range. Consequently, patient characteristics at the extreme ends of these parameters are not represented in the dataset. Variations in other factors, such as urine conductivity, were not considered in this analysis. Future studies should develop a library of high-fidelity 3D torso models that incorporate additional tissue domains. Moreover, electrode configurations, particularly multi-row arrangements, should be simulated to more closely approximate in vivo placement patterns, enabling direct comparison between computational and experimental results.

## 5. Conclusions

This study investigated the influence of patient characteristics on two features commonly used in BIA and EIT through computer modeling. We provided reference signal ranges for waist circumferences and subcutaneous fat thicknesses, which can be used to augment existing datasets and guide AFE design to ensure sufficient hardware resolution across a broad patient population. Our results demonstrate that generalized models can eliminate the need for cumbersome patient-specific calibrations, such as requiring patients to void into a commode for a volume ground truth, while still achieving performance comparable to individualized approaches. These contributions support the development of more robust sensing hardware and bladder volume estimation algorithms applicable to diverse patient cohorts.

Future work should expand the anatomical parameter space and enhance modeling fidelity as a next step toward clinical translation. Employing higher-resolution three-dimensional torso models that include additional tissue domains while maintaining a range of adipose tissue thicknesses, waist geometries, and multi-row electrode configurations would improve alignment between simulation and in vivo placement. This approach would enable patient-matched digital twin studies to develop transfer functions that better align simulated results with in vivo measurements. Incorporating these models may further refine generalized predictors, reinforce hardware design requirements, and facilitate direct comparison to clinical datasets as wearable bladder-monitoring systems advance.

## Figures and Tables

**Table 1:** COMSOL swept parameters.

			Range		
Variable	Source	Unit	Min	Max	Step
Bladder	[49]	m L	10	460	-
Fat	[48]	m	0.02	0.08	0.02
Frequency		k Hz	10	250	5/decade
Waist	[47]	m	0.775	1.275	0.10

**Table 2:** Simulation domains’ dielectric properties.

Sim. Domain	Source Tissue	Parameterization	Source
Bladder	Urine	4-Cole-Cole	[53]
Electrode	EKG Gel	Piecewise Cubic	[51]
Fat	Avg. Infiltrated Fat	4-Cole-Cole	[52]
Muscle	Muscle	4-Cole-Cole	[52]
Skin	Dry Skin	4-Cole-Cole	[52]

**Table 3:** Model Training Parameters.

Model	Data	Type	Training Features	Cross Validation	Folds
1	|ΔV|	Patient Specific	90	LOO	5
2	|ΔV| + PF	Generalized	92	K-fold	15
3	|VCR|	Patient Specific	90	LOO	5
4	|VCR| + PF	Generalized	92	K-fold	15

PF = Patient (Characteristic) Features.

**Table 4:** Baseline Model Cross Validation Results.

	MAE							MAPE						
M	$\bar{x}$	σ	$F_{\bar{x}}$	$F_{\sigma }$	$F_{\bar{x},N}$	$\bar{J}$	S	$\bar{x}$	σ	$F_{\bar{x}}$	*F_σ_*	$F_{\bar{x},N}$	$\bar{J}$	S
1	6.89	3.44	8.67	3.13	0.03	0.44	0.07	3.62	1.79	49.07	11.16	0.16	0.78	0.76
2	28.84	12.14	13.93	1.44	0.11	0.89	0.88	41.40	25.36	13.93	1.44	0.11	0.89	0.88
3	1.65	0.75	90.00	0.00	1.00	1.00	1.00	0.78	0.43	16.87	7.57	0.39	0.40	0.10
4	7.91	5.80	43.27	3.71	0.99	0.98	0.97	4.44	2.20	58.20	6.62	1.00	0.98	0.98

The first column denotes the model number. The MAE $\bar{x}$ & σ are expressed in mL, MAPE $\bar{x}$ & σ are expressed as a percent, *F* refers to the number of features, $F_{\bar{x},N}\in \left[0,1\right]$ the proportion of features used from the neighboring method set, and stability, labeled as S, is the fraction of selected features that are in the set ${\hat{S}}^{\mathrm{stable}}$.

**Table 5:** Optimal Elastic Net Hyperparameters From Cross Validation.

	MAE		MAPE	
Model	α	$L_{1-\mathrm{ratio}}$	α	$L_{1-\mathrm{ratio}}$
1	1.00 $\times \;10^{-7}$	1.00	1.06 $\times \;10^{-6}$	0.90
2	1.00 $\times \;10^{-7}$	1.00	1.00 $\times \;10^{-7}$	1.00
3	1.00 $\times \;10^{-7}$	0.10	3.07 $\times \;10^{-4}$	0.70
4	2.89 $\times \;10^{-5}$	1.00	1.51 $\times \;10^{-4}$	0.10

**Table 6:** Generalized Model Performance With Scaled Inputs.

			MAE		MAPE	
Model	$F_{\mathrm{set}}$	Scaling	$\bar{x}$	σ	$\bar{x}$	σ
2	MAE	Linear	35.49	15.39	24.17	14.53
2	MAE	Exp.	8.78	4.84	9.31	4.31
2	MAPE	Linear	35.49	15.39	24.17	14.53
2	MAPE	Exp.	8.78	4.84	9.31	4.31
4	MAE	Linear	12.53	10.45	10.23	6.49
4	MAE	Exp.	**5.67**	**4.17**	**3.91**	**1.70**
4	MAPE	Linear	11.18	9.38	10.21	6.41
4	MAPE	Exp.	**5.44**	**3.09**	**4.38**	**2.80**

$F_{set}$ is the elastic net selected features for a given scoring criterion, scaling refers to the method used to process the frames, MAE $\bar{x}$ & σ are expressed in mL, and MAPE $\bar{x}$ & σ are expressed as a percent. Bolded values indicate performance exceeding at least one of the patient specific models in Table 4.

## Data Availability

Generated simulation data from this study is not available for sharing due to industry sponsorship, which has resulted in proprietary work. Code used publicly available data analysis techniques and regression algorithms detailed in Section 2.

## Supplementary Materials

The following supporting information can be downloaded at https://www.mdpi.com/article/10.3390/s25247635/s1: Figure S1: An annotated, semitransparent COMSOL model showing the current flow as yellow lines between current-carrying electrodes 0 and 5. Electrode 0 in red and electrode 5 in blue show the high and low electrical potentials, respectively. The electrodes are centered on patches of skin, significantly lowering degrees of freedom. Table S1: Dielectric material sources and conductivities at 50 kHz. Table S2: Patient simulations after impossible configurations removed. Table S3: ΔV statistics by bladder volume. Note the baseline is Vol_0_ = 10 mL. Table S4: *VCR* statistics by bladder volume. Note the baseline is Vol_0_ = 10 mL. Figure S2: Scores over cross-validation (CV) grid search for optimal elastic-net parameters. There are 10 lines indicating the 0.1 to 1.0 in 0.1 steps of the L1 ratio parameter, with 1.0 being the darkest. The optimal values, determined by the scorer, that were selected most often during CV are displayed on the graphs.
