# Supplementary material for: Towards Generalized Bioimpedance Models for Bladder Monitoring: The Role of Waist Circumference and Fat Thickness
Source: Sensors (Basel). 2025 Dec 16;25(24):7635. doi: 10.3390/s25247635 (PMC12736868; doi:10.3390/s25247635)
Supplement: Supplementary file 1 [file sensors-25-07635-s001.zip › sensors-3990719-supplementary.pdf]

Supplementary Material for Towards Generalized Bioimpedance Models for Bladder Monitoring: The Role of Waist Circumference and Fat Thickness

December 6, 2025

List of Tables

1 Dielectric material sources and conductivities at 50 kHz. 2
2 Patient simulations after impossible configurations removed. 3
3 ΔV statistics by bladder volume. Note the baseline is $\text{Vol}_0 = 10\,\text{mL}$. 4
4 VCR statistics by bladder volume. Note the baseline is $\text{Vol}_0 = 10\,\text{mL}$. 4

List of Figures

1 An annotated, semitransparent COMSOL model showing the current flow as yellow lines between current-carrying electrodes 0 and 5. Electrode 0 in red and electrode 5 in blue show the high and low electrical potentials, respectively. The electrodes are centered on patches of skin, significantly lowering degrees of freedom. 1
2 Scores over cross-validation (CV) grid search for optimal elastic-net parameters. There are 10 lines indicating the 0.1 to 1.0 in 0.1 steps of the L1 ratio parameter, with 1.0 being the darkest. The optimal values, determined by the scorer, that were selected most often during CV are displayed on the graphs. 5

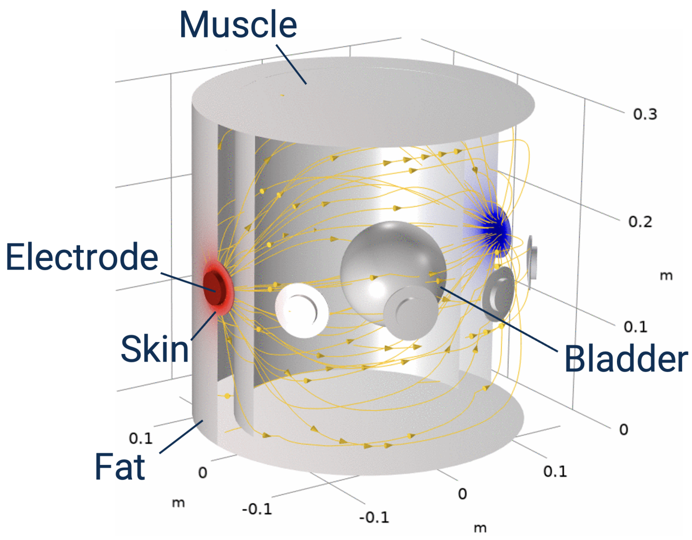

Figure S1: An annotated, semitransparent COMSOL model showing the current flow as yellow lines between current-carrying electrodes 0 and 5. Electrode 0 in red and electrode 5 in blue show the high and low electrical potentials, respectively. The electrodes are centered on patches of skin, significantly lowering degrees of freedom.

Table S1: Dielectric material sources and conductivities at 50 kHz.

Simulation Domain	Dielectric Material Source	σ @ 50 kHz
Bladder	Urine	1.750
Electrode	Gel EKG Electrodes	0.180
Fat	Avg. Infiltrated Fat	0.043
Muscle	Muscle	0.352
Skin	Dry Skin	2.73×10^{-4}

The parameterized functions were evaluated at 50 kHz, the primary frequency used throughout the volume estimation part of our study. These values are provided to facilitate rapid reproduction of our results. Full functional forms can be found in [1]–[4].

Table S2: Patient simulations after impossible configurations removed.

Simulation ID	Waist (m)	Fat Thickness (m)
0	0.775	0.02
1	0.875	0.02
2	0.875	0.04
3	0.975	0.02
4	0.975	0.04
5	1.075	0.02
6	1.075	0.04
7	1.075	0.06
8	1.175	0.02
9	1.175	0.04
10	1.175	0.06
11	1.275	0.02
12	1.275	0.04
13	1.275	0.06
14	1.275	0.08

Supplementary Note: Probable Model Configurations

For each stimulation frequency, the simulation framework produced 8,100 datapoints, corresponding to 90 measurement frames, evaluated across 15 virtual patients and 6 bladder volumes. Each datapoint represents the steady-state electrode voltage differential extracted from COMSOL via domain probes.

During automated parameter sweeps, certain geometric combinations occasionally caused the expanding bladder domain to intersect the subcutaneous fat layer. Such cases are anatomically unrealistic. These invalid configurations were automatically excluded from the aggregated dataset. The surviving models therefore represent physically plausible anatomies spanning the majority of the empirical range of waist circumferences, fat thicknesses, and bladder volumes evaluated in this study.

A complete listing of the surviving simulation cases, including their associated waist circumference, fat thickness, bladder volume, and frequency parameters, is provided in Supplementary Table 2. This table forms the final dataset used for all analyses presented in the main text.

Table S3: ΔV statistics by bladder volume. Note the baseline is $\text{Vol}_0 = 10$ mL.

Volume (mL)	Feature	Pearson's r	p-value	Slope	Intercept	R^2
0.020	Waist	-0.601	0.0004	-1.397e-04	1.918e-04	0.362
0.020	Fat	-0.150	0.0004	-2.967e-04	5.121e-05	0.022
0.020	Freq	-0.044	0.0004	-2.622e-08	4.128e-05	0.002
0.100	Waist	-0.602	0.0004	-7.101e-04	9.761e-04	0.363
0.100	Fat	-0.154	0.0004	-1.553e-03	2.633e-04	0.024
0.100	Freq	-0.045	0.0004	-1.346e-07	2.112e-04	0.002
0.220	Waist	-0.601	0.0004	-1.496e-03	2.063e-03	0.361
0.220	Fat	-0.161	0.0004	-3.415e-03	5.671e-04	0.026
0.220	Freq	-0.045	0.0004	-2.891e-07	4.521e-04	0.002
0.340	Waist	-0.597	0.0004	-2.190e-03	3.033e-03	0.357
0.340	Fat	-0.166	0.0004	-5.206e-03	8.509e-04	0.028
0.340	Freq	-0.046	0.0004	-4.331e-07	6.752e-04	0.002
0.460	Waist	-0.593	0.0004	-2.791e-03	3.884e-03	0.351
0.460	Fat	-0.171	0.0004	-6.884e-03	1.113e-03	0.029
0.460	Freq	-0.047	0.0004	-5.668e-07	8.806e-04	0.002

Table S4: VCR statistics by bladder volume. Note the baseline is $\text{Vol}_0 = 10\,\text{mL}$.

Volume (L)	Feature	Pearson's r	p-value	Slope	Intercept	R^2
0.020	Waist	-0.486	0.0004	-2.962e+00	4.111e+00	0.237
0.020	Fat	-0.303	0.0004	-1.575e+01	1.496e+00	0.092
0.020	Freq	-0.004	0.6391	-6.879e-05	8.912e-01	0.000
0.100	Waist	-0.494	0.0004	-1.476e+01	2.055e+01	0.244
0.100	Fat	-0.316	0.0004	-8.057e+01	7.607e+00	0.100
0.100	Freq	-0.005	0.6123	-3.550e-04	4.513e+00	0.000
0.220	Waist	-0.501	0.0004	-3.038e+01	4.257e+01	0.251
0.220	Fat	-0.334	0.0004	-1.729e+02	1.620e+01	0.111
0.220	Freq	-0.005	0.5907	-7.807e-04	9.559e+00	0.000
0.340	Waist	-0.505	0.0004	-4.358e+01	6.152e+01	0.255
0.340	Fat	-0.351	0.0004	-2.588e+02	2.410e+01	0.123
0.340	Freq	-0.005	0.5731	-1.181e-03	1.416e+01	0.000
0.460	Waist	-0.508	0.0004	-5.446e+01	7.750e+01	0.258
0.460	Fat	-0.369	0.0004	-3.380e+02	3.131e+01	0.136
0.460	Freq	-0.006	0.5311	-1.579e-03	1.833e+01	0.000

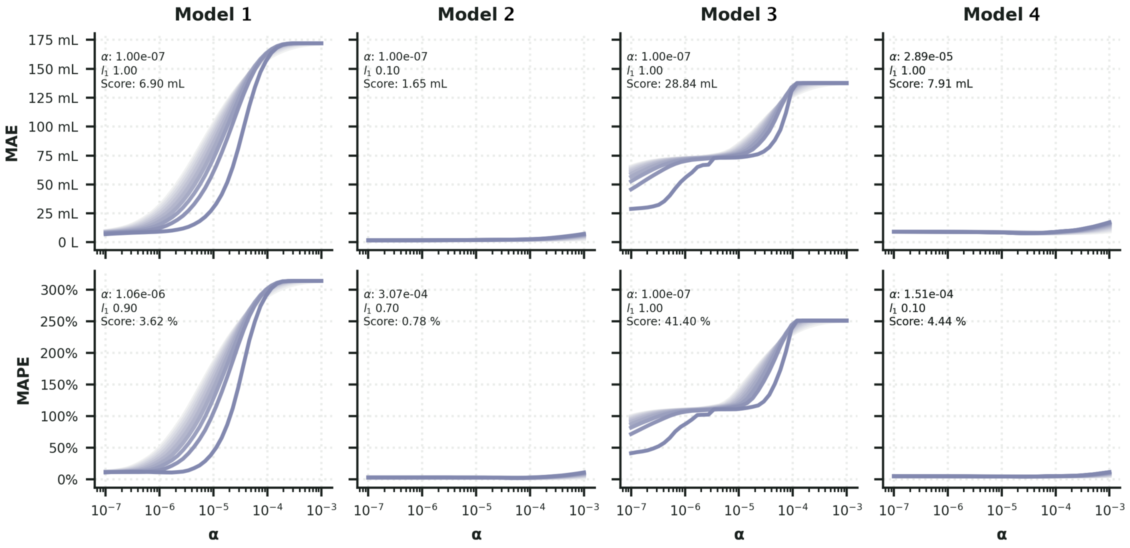

Figure S2: Scores over cross-validation (CV) grid search for optimal elastic-net parameters. There are 10 lines indicating the 0.1 to 1.0 in 0.1 steps of the L1 ratio parameter, with 1.0 being the darkest. The optimal values, determined by the scorer, that were selected most often during CV are displayed on the graphs.

References

[1] IT'IS Foundation, *Tissue Properties Database V4.1*, 2022. DOI: 10.13099/VIP21000-04-1. [Online]. Available: https://itis.swiss/virtual-population/tissue-properties/downloads/database-v4-1/ (visited on 09/21/2023).

[2] C. Gabriel, "Compilation of the Dielectric Properties of Body Tissues at RF and Microwave Frequencies," Occupational and Environmental Health Directorate, Radiofrequency Radiation Division, Brooks Air Force Base, Texas (USA), Tech. Rep. Al/OE-TR-1996-0004, 1996. [Online]. Available: https://apps.dtic.mil/sti/citations/ADA303903.

[3] A. Peyman and C. Gabriel, "Dielectric properties of porcine glands, gonads and body fluids," *Physics in Medicine and Biology*, vol. 57, no. 19, N339–N344, Oct. 2012, ISSN: 0031-9155, 1361-6560. DOI: 10.1088/0031-9155/57/19/N339. [Online]. Available: https://iopscience.iop.org/article/10.1088/0031-9155/57/19/N339 (visited on 10/21/2024).

[4] F. Lin, Z. Wei, J. Yang, *et al.*, "A Comparative Study of the Electrodes Gels' Electrical Properties in the Measurement Issues of Intrabody Communication," *Beijing Institute of Technology*, vol. 31, no. 1, pp. 71–80, Feb. 2022. DOI: 10.15918/j.jbit1004-0579.2021.085. [Online]. Available: http://journal.bit.edu.cn/jbit/en/article/doi/10.15918/j.jbit1004-0579.2021.085.
